# Lessons from workplace health promotion efforts in Thailand

**DOI:** 10.1016/j.lansea.2024.100393

**Published:** 2024-03-21

**Authors:** Jidapa Hanvoravongchai, Chathaya Wongrathanandha, Piya Hanvoravongchai

**Affiliations:** a Department of Preventive and Social Medicine, Faculty of Medicine, Chulalongkorn University, 1873 Rama IV Rd, Pathum Wan, Bangkok, Thailand; b Department of Community Medicine, Faculty of Medicine Ramathibodi Hospital, Mahidol University, 270 Rama VI Rd, Thung Phaya Thai, Ratchathewi, Bangkok, Thailand

In Thailand, there have been significant efforts, both at the organization level and at national level, to foster stronger health promotion in the workplace. Several workplace health promotion (WHP) programs have been implemented at the national level, and several initiatives launched by the entities under the Ministry of Public Health (MOPH). The Table 1 below shows the major national programs in Thailand. Their objectives align with the National Health Reform agenda, which aims to actively enhance health among Thai workers.^1^ Despite these efforts, WHP in Thailand is still limited,^2^^,^^3^ and the country continues to face a rising health burden of non-communicable diseases.^4^ While 60–80% of enterprises reported having health promotion policy in the workplace, the majority are large scale enterprises or those in the industrial area,^5^, ^6^, ^7^ with limited engagement of small to medium enterprises.

**Table 1:** List of national level WHP programs in the study.

Program name	Responsible agencies
Happy workplace	Thai Health Promotion Foundation
Healthy organization	Raipoong (No tummy) Academy
Disease and Hazard-Free Enterprises with Physical and Mental Well-being	MOPH Department of Disease Control, Division of Occupational and Environmental Diseases
Wellness center	MOPH Department of Disease Control, Division of Occupational and Environmental Diseases
Healthy workplace, Happy for life	MOPH Department of Health, Bureau of Environmental Health
“10 Packages” program	MOPH Department of Health, Bureau of Health Promotion

To understand the challenges facing national effort to promote more workplace health promotion, we conducted an extensive review and key informant interviews on these six national-level programs. Our findings revealed four important lessons on the factors contributing to the development of health promotion in the workplace.

First, successful WHP requires strong engagement from all actors involved in the welfare and productivity of workers. The Ministry of Labour and the Ministry of Industry are key government agencies responsible for most formal sector employment in Thailand. However, they have not invested adequately in health aspect. Their roles are limited to be mainly in the public relations of the programs, while MOPH's personnel at provincial/regional levels are the main implementors of the interventions. It is imperative for the MOPH to work beyond health sector and engage these two ministries as well as other private stakeholders, such as the Federation of Thai Industries and the Thai Chamber of Commerce. These collaborations can lead to joint funding initiatives and resource pooling to support a common goal of improving employees' health.

Second, the lack of coordination between different departments of the MOPH and between the MOPH and other organizations is a major shortcoming. While the MOPH is the national agency responsible for health, a number of divisions and departments have independently initiated their own programs without a clear coordination, resulting in numerous programs with similar policies and activities. This causes confusion among provincial teams implementing the programs with participating enterprises. Suggestions to harmonize these programs in the past had not been successful due to overlapping roles of each division, but it does not mean they should continue as is. The MOPH could define clear roles for each division and develop a harmonized policy, including a unified framework and evaluation criteria. National health reform movement is also a window of opportunity to unite all key players together for synergistic approaches towards a better WHP.

Third, even though all six WHP support programs engage with employers and provided technical assistance to strengthen their capacity to deliver customized WHP activities, they fail to emphasize effective feedback mechanisms to collect and use information on health improvement and productivity gains to support enterprises' program evaluation and improvement. For example, none of the WHP programs promote the use of health screening data collected from annual checkups to support WHP efforts. At the enterprise level, some enterprises do not have questionnaires or formal assessment to evaluate health outcomes and productivity gains. Establishing an effective health information exchange between relevant public organizations, such as Social Security Office and the Department of Labour Protection and Welfare, could improve the effectiveness and efficiency of these programs by facilitating targeted health promotion interventions tailored to each enterprise's specific challenges.

Fourth, new policy tools or interventions are necessary to shape employers and employees' behaviours. Four of the six national programs conducted performance evaluation of participating enterprises and provided certificates as rewards. Such recognition for enterprises already committed to health promotion is a weak incentive for others. Stronger financial policy by the relevant ministries, such as tax credits or a reduction in Social Security's Workmen Compensation Fund contribution, could be effective in encouraging broader program participation and facilitating more impactful WHP interventions. Countries like France, Germany, Italy, the United Kingdom, and Singapore have successfully provided tax credits related to health and well-being as one of their national level policies.^8^, ^9^, ^10^

In conclusion, national level WHP programs could be more effective through enhanced collaboration among organizations, leveraging on health information, and implementing new policies and incentives. The creation of harmonized national level WHP policies, new form of financial incentives and support, with better intelligence systems are necessary to incentivize stronger workplace health promotion practices in Thailand.

## Contributors

JH and PH were involved in the initial design of the study. JH and CW developed the interview questionnaire and drafted the report. JH were responsible for data collection and drafted the initial manuscript, and all the authors contributed to its development and approved the final version.

## Data sharing statement

The data that support the findings of this study are available on request from the corresponding author from the date of publication.

## Declaration of interests

All authors have no conflicts of interest regarding this study.
